# Functional Nanoparticles for Tumor Penetration of Therapeutics

**DOI:** 10.3390/pharmaceutics10040193

**Published:** 2018-10-18

**Authors:** Yu-Lin Su, Shang-Hsiu Hu

**Affiliations:** Department of Biomedical Engineering and Environmental Sciences, National Tsing Hua University, Hsinchu 30013, Taiwan; s104012902@m104.nthu.edu.tw

**Keywords:** drug delivery, controlled release, nanomedicine, functional materials, composites

## Abstract

Theranostic nanoparticles recently received great interest for uniting unique functions to amplify therapeutic efficacy and reduce side effects. Despite the enhanced permeability and retention (EPR) effect, which amplifies the accumulation of nanoparticles at the site of a tumor, tumor heterogeneity caused by the dense extracellular matrix of growing cancer cells and the interstitial fluid pressure from abnormal angiogenesis in the tumor inhibit drug/particle penetration, leading to inhomogeneous and limited treatments. Therefore, nanoparticles for penetrated delivery should be designed with different strategies to enhance efficacy. Many strategies were developed to overcome the obstacles in cancer therapy, and they can be divided into three main parts: size changeability, ligand functionalization, and modulation of the tumor microenvironment. This review summarizes the results of ameliorated tumor penetration approaches and amplified therapeutic efficacy in nanomedicines. As the references reveal, further study needs to be conducted with comprehensive strategies with broad applicability and potential translational development.

## 1. Introduction

Theranostic nanoparticle is a developing field of nano-medicine which combines therapy and diagnostic approach into one single agent in nanoscale by applying one or more than one material. For precision medicine of cancer therapy, theranostics offers a transition from traditional chemotherapy to a contemporary personal and precision medicine treatment. By integrating the functional materials, theranostic nanoparticles can unite diagnostic and therapeutic applications effectively, allowing for imaging, diagnosis, controlled drug release and monitoring.

**Figure 1:**
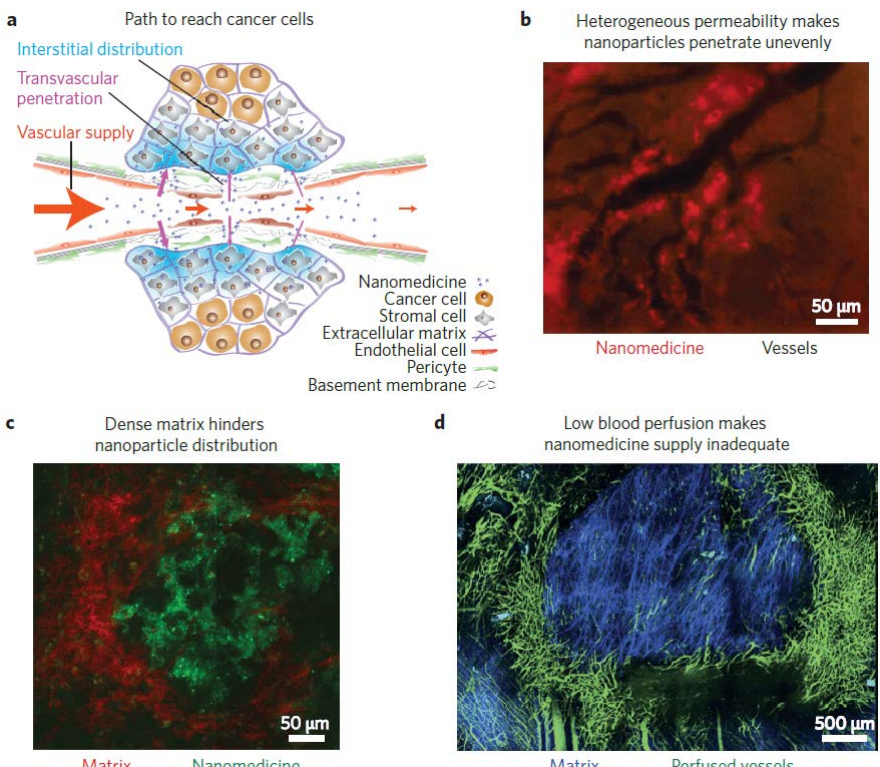
The barriers of functional nanoparticles for penetrated delivery into the tumor. (**a**) The path to the cancer cells. (**b**) Heterogeneity of the tumor decreases the penetration of drug/nanoparticles unevenly. (**c**) The dense extracellular matrix hinders nanoparticle distribution. (**d**) Low blood perfusion causes the nanomedicine supply to be insufficient. Reprinted from Reference [3] with permission from Springer Nature, 2013.

Traditional strategies, including surgical resection, radiotherapy, and chemotherapy, either alone or in combination, are currently employed for cancer treatment. However, these approaches usually target or treat the tumor nonspecifically and exhibit limited therapeutic efficacy. Recently, advances in nanotechnology led to the development of numerous new formulations for antitumor therapy to reduce side effects and to improve therapeutic efficacy. Some of the nanoformulations such as Abraxane^®^, Onivyde^®^, Marqibo^®^, and Nanotherm^®^ were approved by the Food and Drug Administration (FDA) and are applied in clinics [1]. These nanoparticles transport the therapeutic agents to the tumor via the enhanced permeability and retention (EPR) effect and passively accumulate at the tumor site. Through this mechanism, however, most particles accumulate at the periphery of the tumor, and only a small fraction of the injected therapeutic agents penetrates the deep tumor due to the heterogeneity of tumors, leading to the limited life time of the patient [2,3] (Figure 1). The heterogeneity of tumors is attributed to the dense extracellular matrix (ECM) and interstitial fluid pressure (IFP), because the rapid proliferation of tumor cells leads to reduced vascular density and irregular blood flow. Furthermore, the compression of blood and lymphatic vessels is also induced by the proliferating cancer cells. The composition and structure of the ECM result in the formation of hypoxic, weakly acidic, or nutrient-stripped tumor microenvironments, which not only decelerate the movements of the therapeutic agents, but also potentially reduce the activities of the anticancer agents. Moreover, the interactions between cancer cells far from the blood vessels and the therapeutic agents are lowered since the tumor barriers block their transportation [4]. In addition, vessel abnormalities, fibrosis, and contraction in the tumor matrix can also increase the IFP, lower the transcapillary transport, and decrease the internalization of drugs in the tumor [5]. These obstacles form critical barrier walls that obstruct nanomedicines from penetrating deep into the tumors, and accumulation around the perivascular region is usually observed [6].

**Figure 2:**
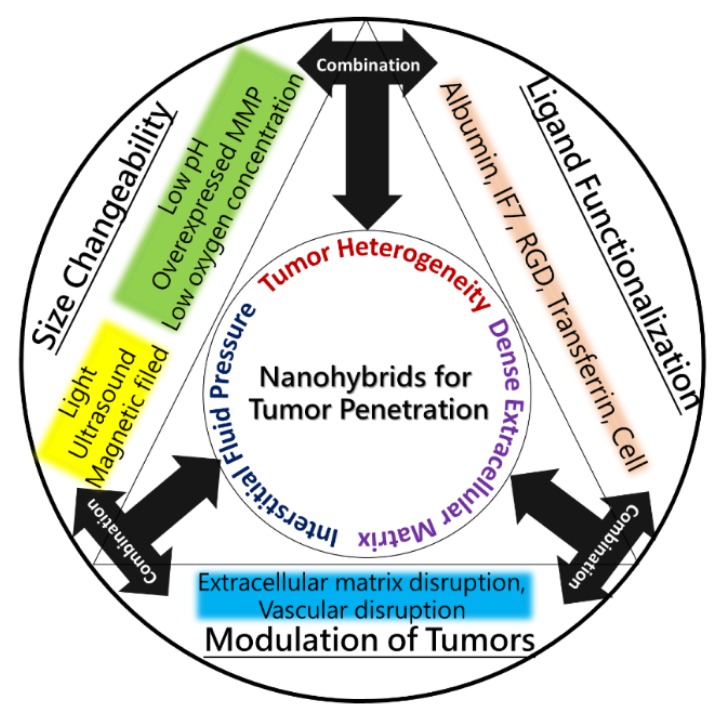
The scheme of different strategies of functional nanoparticles for the penetrated delivery of drugs and energy molecules in recent years.

Several studies reported the ability to overcome these barriers in tumor treatments. Specifically, the treatments can be divided into three strategies involving size, functionalization, and modulation. Firstly, the size effect is a passive but extremely important and useful factor to control the penetrating ability of nanomedicines. Through the size-responsiveness to inner stimuli such as pH, oxygen concentration, and overexpressed proteins in the tumor microenvironment, or to outer stimuli such as light, ultrasound, and magnetic fields, the conformations of the nanoparticles can be varied to enhance their distribution at the tumor site. Secondly, functionalization is an active, efficient, and continuous process of penetrating ligands or structures for transcytosis and transvascular deliveries. Thirdly, the modulation of tumors is also an effective process to vary the tumor microenvironment and decrease the barriers of tumor heterogeneity. The dense ECM of the tumor impedes the drugs/nanoparticles from entering deep into the tumor, leading to insufficient therapeutic efficacy. Therefore, modulating tumors to loosen their ECM can improve the distribution of drugs and nanoparticles at the tumor (Figure 2). To evaluate the penetration of nanoparticles, researchers developed different kinds of models, including three-dimensional (3D) multitumoral spheroids (MTS) and an extracellular matrix model, to prove the tumor penetration [7]. This review integrates the physicochemical properties of different nanomaterials and the pharmacological strategies for enhancing their penetration, and a great step forward is expected in overcoming the limitations of nanomedicines in tumor therapies and clinical trials.

## 2. Tumor Penetration by Size Changeability 

According to the size effect, many studies focused on stimulus-triggering (outer impact) or the tumor microenvironment (inner impact) to change the sizes of the nanomedicines. Tumor-penetrating ability was proven to be inversely proportional to the sizes of the nanoparticles. For example, Tang et al. revealed that monodisperse 50-nm silica nanoconjugates can penetrate into the tumor more deeply than 200 nm particles [8]. In addition, Chauhan et al. demonstrated that 12-nm nanoparticles cross into the tumor better than larger nanoparticles, and both diffusive and convective modes of penetration of 12-nm particles in transvascular and interstitial tissues are faster than those of larger particles [9]. The comprehensive experiment was conducted by Cabral et al., who showed the accumulated efficacy of polymeric micelles with sizes ranging from 30 to 100 nm in the tumors. Most polymeric micelles can be observed in highly permeable tumors and show an antitumor efficacy. However, only 30-nm micelles can accumulate in impermeable tumor tissues [10,11]. The treatments and applications for tumor penetration based on the size effect are described below with different classifications.

### 2.1. Stimulus-Triggering (Outer Factors)

Stimulus-triggering strategies were developed for each kind of conventional therapy, such as hyperthermia, dynamic therapy, and synergistic chemotherapy. Apart from that, the size should be suitable for EPR effect; many researchers also concentrated on whether these stimulus-triggering methods could facilitate a size change and enhance tumor penetration. Therefore, we discuss three main sections: light, ultrasound, and magnetic fields. Each section discusses how well-developed functional nanoparticles contribute to penetrated delivery into solid tumors.

#### 2.1.1. Light

In conventional therapies, light can be used in photothermal, photodynamic, and photoacoustic applications since the specific nanomaterials can absorb various wavelengths of irradiated light. After the light treatment, energy from light converts to heat or electrons and is able to change the properties of nanomaterials, such as temperature, surface hydrophobicity, morphology, or chemical reactivity. Thus, many researchers applied light to control particle sizes to enhance drug distribution. Tong et al. exhibited that spiropyran nanoparticles sensitively shrink from 103 to 49 nm upon ultraviolet (UV) light irradiation. UV light allows the hydrophobic spiropyran to change to zwitterionic merocyanine, which is smaller in size, and varies the physical assembly properties. Additionally, because merocyanine is less stable than spiropyran, merocyanine nanoparticles spontaneously return to spiropyran nanoparticles under visible light or darkness with an increase in size. Such reversible photoswitching of spiropyran nanoparticles enhances tumor penetration and promotes drug delivery into the tumor [12,13]. Our group also exploited a photoresponsive pea-like nanocapsule that can transport multiple small polyvinyl alcohol micelles fully loaded with the hydrophobic drug docetaxel. A photopenetrative delivery to the tumor site is triggered by near-infrared (808 nm) irradiation that is able to rupture the structures of the embedded micelles inside the capsule and release them. This trigger also gives rise to thermal ablation of the tumor and enhances the permeability of small micelles, thus facilitating drug penetration into the tumor [14].

#### 2.1.2. Ultrasound

Ultrasound-responsive nanoparticles are used in either tumor treatment or imaging. With the excellent advancement, ultrasound received much attention as a powerful drug-delivery approach because it can focus deep into the organs and trigger gas generation to allow drug release in certain sites. Ultrasound-responsive nanoparticles steadily progressed from unstable large-sized particles (100 to 300 μm) to smaller particles ranging from 1 to 8 μm, which can cross the tumor barrier for image-guided drug delivery. For example, Min et al. presented carbonate copolymer nanoparticles that can generate CO_2_ gas through a CO_2_-generating approach by applying the carbonate copolymer precursor. The description was modified. It reads: “These gas-generated nanoparticles at the tumor can be hydrolyzed to form large numbers of CO_2_ bubbles with micrometers that efficiently absorb ultrasound at the specific site and timing for ultrasound (US)-actuated drug delivery” [15]. In addition, Wang et al. opened up an ultrasound-responsive liposome as a nanocarrier containing an air layer within a lipid bilayer. The anticancer doxorubicin-loaded Polyamidoamine (PAMAM) dendrimers and nitric-oxide-donating nitrosoglutathione were embedded in the nanocarrier. Once the particles were trapped in the tumor, ultrasound triggered the gas layer of the particle to rupture the lipid bilayers and release the embedded drugs/dendrimers into the tumor vasculature, extravasating the endothelial junction deep into the tumor. In the meantime, the cell membranes of the tumor interacted with the PAMAM dendrimers to enhance cellular internalization. The therapeutic efficacy of doxorubicin was then proven by tumor penetration with ultrasound stimulation [16].

#### 2.1.3. Magnetic Fields

Magnetic nanoparticles such as superparamagnetic iron-oxide nanoparticles (SPION) are well-developed for hyperthermia, drug delivery, and image guiding. As an alternating magnetic field is applied, magnetic nanoparticles vibrate according to the direction of the magnetic field, which can increase the surrounding temperature. Therefore, researchers utilized this property to improve tumor penetration combined with hyperthermia or drug delivery. Kong et al. designed anticancer drug-entrapped magnetic capsules that can be regulated through an external gradient magnetic field for tumor-targeting and penetration. Furthermore, these capsules can reach deep into the tumor and exhibit a switchable drug release through an external alternating magnetic field [17]. In addition, Soheilian et al. applied an external magnetic field to induce the aggregation of 150-nm magnetic nanoparticles into sizeable assemblies. Under a dynamic magnetic field, the switchable magnetic field direction can cause the particles to group into an attractive or repulsive configuration, promoting the particle clusters to assemble or disassemble. Thus, this reverse property was utilized to create a simple analytical model to evaluate the kinetic thresholds of the assembly and disassembly of magnetic nanoparticles, and these methods demonstrated good tumor penetration into a gel with a similar pore size as that of tumor tissue [18].

### 2.2. Tumor Microenvironment (Inner Factors)

Unlike normal tissue, the tumor microenvironment contains overexpressed proteins (matrix metalloproteinase (MMP) groups) and has a low pH value (6.5–6.8) and low oxygen concentration (hypoxia). It is easy for functional nanoparticles to sense the tumor microenvironment and change their conformation. Meanwhile, size and morphology can also be adapted to enhance tumor penetration. Thus, diverse nanoparticles were manufactured with size switchability to increase their ability to reach deep into the tumor.

#### 2.2.1. Overexpressed MMP

One of the most broadly evaluated and overexpressed proteinases in tumors is the MMP family, including MMP, MMP-2, and MMP-9. The MMP family can selectively recognize and cleave the peptide sequence, Pro–Leu–Gly–Leu–Ala–Gly, which can be functionalized on nanoparticles. The strategy is that, as the nanoparticles arrive at the tumor sites, the overexpressed MMP cuts the binding of the peptide sequence and disassembles the nanoparticles, whose decreased size enhances tumor penetration. Through this concept, Wong et al. developed a multistage system in which 10-nm quantum dot (QD) nanoparticles were embedded in 100-nm gelatin nanoparticles. The in vivo circulation time was extended by the gelatin/QD nanoparticles, and strong accumulation was exhibited at the targeted tumor sites. As the nanoparticles extravasated from the leaky areas of the tumor blood vessel, the nanoparticles shrank to 10 nm in response to overexpressed MMP-2 in the tumor environment, which improved the penetration ability in the matrix. The 10-nm model nanoparticles proved that carriers with this function are potentially useful for therapeutics as the particles become small and can penetrate and deliver drugs deep into the tumor [19]. Additionally, Ruan et al. also developed shrinkable gelatin nanoparticles loaded with small gold nanoparticles and the anticancer drug doxorubicin. Firstly, the shrinkable nanoparticles accumulated at the tumor tissue by the EPR effect had a broad distribution of particle sizes. The shrinkable nanoparticles then penetrated deep into the tumor through the leaky tumor blood vessel, actuated by the degrading gelatin particles via MMP-2, which allowed small-size gold nanoparticles and the anticancer drug doxorubicin to be released. This strategy exhibited good transvascular and interstitial penetration, facilitating the accumulation of particles deep into the tumor and a homogeneous treatment [20].

Tang et al. also developed a tumor-microenvironment-responsive particle embedded with paclitaxel (PTX) and anti-metastatic small interfering RNA (siRNA). The particle is composed of an MMP-cleavable polyethylene glycol (PEG) corona with a cationic shell. The MMP-cleavable PEG ruptures inside the tumor with overexpressed MMP in the tumor microenvironment, promoting the nanoparticle to become positively charged, which improves the efficiency of cellular uptake and the tumor penetration of the drug and siRNA in vivo. Moreover, the weak-acid-actuated drug release in endosomes and lysosomes are carried out via the pH sensitivity of these nanoparticles. The MMP-and pH-responsiveness of these particles can increase the penetration of drugs with size changeability by MMP-cleavable PEG to inhibit tumor growth [21].

#### 2.2.2. Low pH

**Figure 3:**
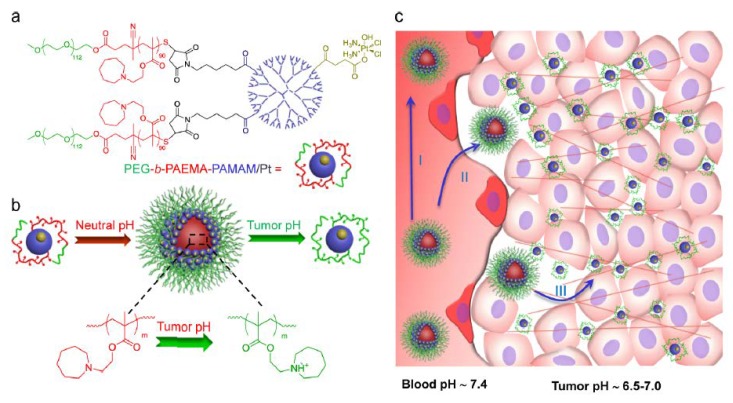
Size-changeable function for tumor penetration. Li et al. reported a tumor-microenvironment-responsive nanoparticle that possessed a switchable size in weakly acidic conditions. (**a**) Structure of drug-loaded polymeric nanoparticles composed of dendrimers and a functional polymer. (**b**) Schematic illustration displaying the functions and self-assembly of polymeric nanoparticles, and pH-sensitive cluster of particles at neutral pH exhibiting a size transition at tumor acidic condition. (**c**) The size transition of drugs/particles overcame tumor barriers in poorly permeable tumor tissue. Reprinted from Reference [23] with permission from the American Chemical Society, 2013.

The tumor microenvironment, where pH is slightly low, is different from normal tissue. Abnormal cell growth causes nutrients to be consumed extremely rapidly and induces the generation of CO_2_, which lowers the pH of the tumor microenvironment. Therefore, functional nanoparticles can be conjugated with pH sensitivity; once they are delivered to the tumors by the EPR effect, they can sense the pH change and then disassemble, shrink, or even switch their size and morphology, which is beneficial for tumor penetration. Sun et al. developed a bioinspired cocoon-like nanoparticle containing a deoxyribonuclease-degradable DNA clew integrated into a pH-sensitive DNase I capsule. Once the particles reached the tumors, the acidic tumor microenvironments actuated the activity of DNase I via shedding of the shells of the capsule, achieving the shelf-degradation of the nanoclew, which promoted the tumor penetration of doxorubicin [22]. In addition, Li et al. also reported a tumor-microenvironment-responsive nanoparticle that possessed a switchable size in weakly acidic conditions. The nanoparticles consisted of amphiphilic polymers containing ionizable tertiary amine groups for real-time pH-responsiveness. Once the particles were accumulated at the tumor, the weakly acidic tumor microenvironment triggered a sharp size transition. Therefore, the drug-loaded dendrimer particles changed in size from 80 nm at neutral pH to less than 10 nm at the tumor, enhancing the tumor penetration and therapeutic efficacy in vivo [23] (Figure 3). Similarly, the same group also reported an intelligent polymeric particle with a rapid size transition after a rapid change in pH. As the particle reached the tumor site, the weakly acidic tumor microenvironment actuated the charged PAMAM dendrimers with a diameter of 5 nm, the efficiency of drug penetration was enhanced, and delivery deep into the tumor was achieved [24,25]. Such spontaneous pH-responsive behavior can enhance the extravasation and accumulation of particles/drugs through the EPR effect, displaying efficient diffusion in the tumor.

#### 2.2.3. Low Oxygen Concentration

Oxygen deficiency, uncontrolled proliferation of cancer cells, inadequate blood flow, and lack of nutrients can generate hypoxia. Hypoxia triggers tumor progression to enhance angiogenesis, epigenetic changes in the cancer cells, and cancer stem-cell production. However, the hypoxic region is located deep inside the tumors and is inaccessible to the carriers through the veins. Therefore, considering the low oxygen concentration in the deep tumor, Kulkarni et al. created lipid nanoparticles (LNs) composed of a hypoxia-sensitive peptide-conjugated lipid. In the presence of low oxygen partial pressure, the LN displayed destabilized the lipid structures and released the embedded drugs, achieving tumor penetration by hypoxia sensitivity [26]. In addition, using redox-sensitivity, Yan et al. developed a robust strategy to manufacture 90-nm nanoclusters that constituted 5-nm lanthanide-doped particles. The peptide/lanthanide nanoclusters simultaneously enhanced the resistance of peptide drugs against proteolysis, and rupture in the environment of the tumor was able to restrain cancer cell growth and promote the tumor penetration of small carriers [27].

## 3. Tumor Penetration by Ligand Functionalization

In tumor treatments, many functionalizations are designed for targeting at a specific site in the tumors. Importantly, nutrients and overexpressed protein transporters attracted great attention for their promising applications in tumor penetration. Therefore, nanoparticles have the ability to reach a certain location in the cancer cells with specific transporters. For example, transcytosis was revealed in many studies to enhance tumor penetration either in the tumor vasculature or in the intratumoral tissue. Moreover, transmembrane and sequential delivery can enhance the distribution of particles in the tumor. We summarized the main functionalizations for tumor penetration and listed them below.

### 3.1. Albumin

Albumin is one of the critical nutrients for the growth of tumors. In normal conditions, albumin is usually not able to enter the brain due to its large molecular size. However, active brain tumors are hungry for nutrients and energy, and the albumin transporter, which uptakes albumin-related compounds into tumor tissues, is highly expressed and can be used for drug delivery and tumor penetration. Lin et al. developed a self-assembled blood–brain barrier (BBB)-penetrating albumin particle with the ability to encapsulate two different hydrophobic drugs (paclitaxel and fenretinide). Albumin-associated proteins, such as secreted proteins that are acidic and rich in cysteine (SPARC), and glycoprotein 60 (gp60) are the two key proteins that regulate the uptake of albumin by tumors through the mechanism of transcytosis across the endothelium and via endocytosis of the tumor. Therefore, albumin-conjugated particles are able to across the BBB to reach the glioma cells through the biomimetic transport of SPARC and gp60. Moreover, with the conjugated cell-penetrating peptide, the albumin-functionalized particle can further enhance tumor penetration and intratumoral infiltration [28].

### 3.2. IF7

Annexin 1 was identified as a biomarker expressed on the tumor endothelium. The expression of annexin 1 in the tumor vasculature was reported in tumors, revealing that it can probably be used as a therapeutic biomarker to target tumor vessels and penetrate tumor tissue. Peptide IF7 (IFLLWQR) is known to specifically bind to annexin 1. Hence, IF7-peptide-conjugated nanoparticles can be a useful tool for enhancing tumor penetration. Yu et al. developed an IF7-conjugated nanoparticle loaded with paclitaxel to improve anti-angiogenic efficacy and the antitumor effect. They evaluated the in vitro endothelium uptake and antiangiogenic behavior of the vasculature of the tumor, the drug-induced apoptosis of the tumor endothelium, and the necrosis of the tumor tissues. While applying the IF7, the nanoparticle significantly enhanced tumor penetration and drug delivery, which inhibited the growth of MCF-7-ADR tumors with a relatively low dose of paclitaxel [29]. Furthermore, Hatakeyama et al. also investigated therapeutic agents of annexin-1-binding peptide as a cancer treatment. IF7 linked to the anticancer drug, SN-38, which inhibits topoisomerase I, injected intravenously into human colon HCT116 tumors bearing nude mice, successfully inhibited the tumor at low doses of drugs without any side effect. These results indicated that IF7 is an excellent targeting ligand that binds to annexin 1 expressed on the surface of tumor vasculature endothelium, and enhances tumor penetration and therapeutic efficacy [30].

### 3.3. Arginine-Glycine-Aspartic Acid (RGD)

**Figure 4:**
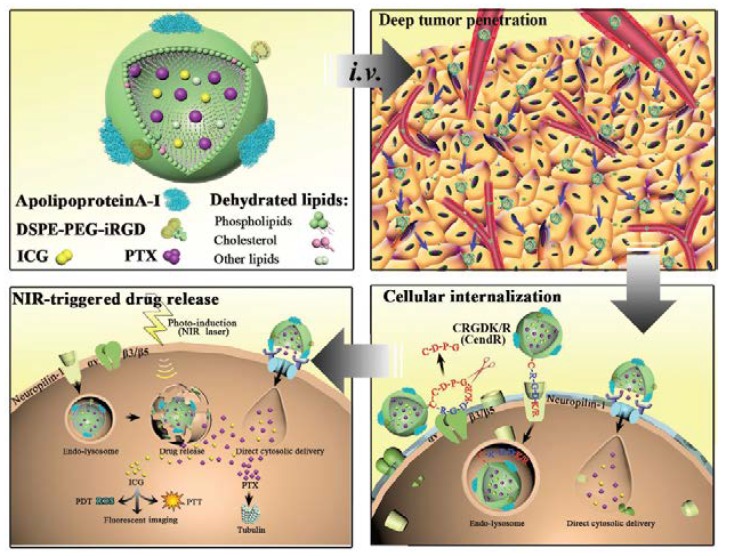
Ligand functionalization for tumor penetration. Wang et al. revealed that after intravenous injection of targeted HDL, iRGD was guided to tumors through three main processes: iRGD targets α_v_ integrins on the endothelium of tumors and undergoes proteolytic cleavage; and subsequently, it achieves tumor penetration. While applying the NIR laser irradiation, rapid drug release is actuated intracellularly, and the photothermal conversion leads to the ROS generation of ICG. Reprinted from Reference [33] with permission from John Wiley and Sons, 2018.

A well-known peptide, arginine-glycine-aspartic acid (RGD), can bind to α_v_β_3_/α_v_β_5_ integrins, which are overexpressed on the endothelial cells of tumor angiogenic vessels and cancer cells in the deep tumor. Binding to the receptor of endothelial cells may increase the targeting capability of nanoparticles to tumor vessels, thereby enhancing the penetration capability of drug delivery systems. Hence, Miura et al. displayed the excellent targeting of U87MG tumors by applying a platinum prodrug encapsulating cyclic RGD (cRGD)-conjugated polymeric micelles. These cRGD-conjugated polymeric micelles exhibited good accumulation at the tumor site and excellent permeability from the blood vessel to the deep tumor, compared to the nontargeted particles. The selective accumulation of cRGD-linked micelles in the tumors occurred after active transcytosis, leading to significant drug penetration and antitumor effects in U87MG human glioblastoma cells [31]. In addition, Gao et al. also enhanced the targeting property of polymeric particles by conjugating RGD and interleukin-13 peptide (IRNP), achieving the targeting of both neovasculature and cancer cells at the same time; RGD was applied for binding α_v_β_3_ on the neovasculature, and interleukin-13 peptide targeted interleukin-13 receptor alpha 2 (IL13Rα2) on the cancer cells. A cell study showed that both targeting ligands enhanced the cellular uptake of C6 and human umbilical vein endothelial cells, suggesting that α_v_β_3_ regulated the internalization of RGD-conjugated particles, and that IL13Rα2 mediated the internalization of interleukin-13 peptide-conjugated particles. The ligand modification led to different endocytosis pathways through modifying the original endocytosis pathways from macropinocytosis to clathrin-mediated endocytosis. On the other hand, the tumor penetration capability of the targeting particles was based on an in vitro tumor spheroid study. It was shown by tissue staining that the targeted particles delivered therapeutic agents and promoted tumor penetration in vivo with higher and more homogeneous intensity than modified nanoparticles [32]. The combination of penetrating ligands was also reported by Wang et al. who formulated bioinspired nanoparticles with native high-density lipoproteins (HDLs) conjugated to iRGD to enhance tumor penetration. The anticancer drug, paclitaxel (PTX), and the fluorescent tracer, indocyanine green (ICG), were loaded into the HDLs for the combination therapy. The iRGD-conjugated HDLs were combined with natural lipoproteins and artificial functions to improve cellular uptake, tumor accumulation, and deep tumor penetration compared to particles that were not modified. Furthermore, the drug-loaded HDLs exhibited a rapid drug release in the cell and achieved chemo-phototherapy that totally suppressed the tumor in vivo [33] (Figure 4). Overall, RGD successfully achieves tumor targeting and tumor penetration, increasing therapeutic efficacy.

### 3.4. Transferrin

Transferrin (Tf) is a globular glycoprotein, and the Tf receptor (TfR) can internalize cancer cells, as well as facilitate the uptake of iron ions. More importantly, TfR is overexpressed on the BBB both in the brain and in the glioma. Therefore, Tf serves as a tumor-targeting ligand and mediates tumor penetration, specifically in gliomas. Kang et al. indicated that binding of the modified cyclic nine-amino peptide, Cys–Arg–Thr–Ile–Gly–Pro–Ser–Val–Cys (CRT), to a complex of TfR on poly(ethylene glycol)-poly(lactic acid) (PEG-PLGA) particles mediated drug delivery through the BBB and penetrated the tumor. The CRT particles exhibited a narrow size distribution on the nanoscale and excellent cellular uptake in the brain capillary endothelial cells and glioma C6 cells via energy-dependent endocytosis. In addition, the authors tested that the delivery of this particle across C6 glioma spheroids was improved, exhibiting excellent therapeutic efficacy in C6 glioma cells. In vivo real-time imaging also revealed that the CRT particles selectively accumulated in the glioma region, especially in the deep brain tumor [34]. Lactoferrin (Lf) is a member of the transferrin family whose receptor is also overexpressed on brain endothelial cells and glioma cells. When Lf is conjugated on the surface of poly(ethylene glycol)-poly(lactic acid) particles, the particles can cross the BBB and the blood−brain−tumor barrier (BBTB). Moreover, a tumor-homing peptide with a C-end rule sequence, tLyP-1, is able to promote tissue penetration via the neuropilin-1-dependent internalization pathway, enhancing the targeting efficiency and tumor penetration. Lf-nanoparticle (Lf-NP) improved cellular association in brain endothelial and C6 glioma cells, boosting cytotoxicity of the anticancer drug (paclitaxel) and tumor penetration both in the 3D glioma spheroids and in the orthotopic brain tumor [35]. The results indicated that the approach of coadministering transferrin-based dual-targeting nanocarriers has excellent potential for anti-glioma drug delivery and tumor penetration.

### 3.5. Cell

Recently, the cell-based delivery system became a new method to enhance tumor penetration by autonomy. Huang et al. demonstrated a hypoxia-targeting delivery system using tumortropic monocytes/macrophages as cellular vehicles to transport C_5_F_12_ molecules and therapeutic agents. Upon exposure to focused ultrasound (FUS), the cell-based delivery system exhibited outstanding capability to kill cancer cells by inducing apoptosis. The immunohistochemical assessment of the tumor sections suggested that the delivery system and therapeutic cargos penetrated into the hypoxic regions of the tumor and were effectively cytotoxic to hypoxic cancer cells. Furthermore, the cell-based delivery system transported the cargo to a distance of 150 mm from the nearest blood vessels after the treatment; in comparison, nontreated particles only penetrated to a depth of approximately 10–15 mm. This work displays the great proof of combining cell-based delivery with an external trigger to transport therapeutic anticancer drugs for enhancing penetrative chemotherapy in the deep tumor [36].

## 4. Tumor Penetration by Modulating the Tumor Microenvironments

### 4.1. Disruption of the Tumor Extracellular Matrix

Reducing the barrier is a promising method for tumor penetration. The dense extracellular matrix hampers the nanoparticles from entering the deep tumor, which results in low therapeutic efficacy and low drug penetration. Thus, to enhance the distribution of drugs, researchers used different design strategies to eliminate the tumor extracellular matrix, such as the use of hyaluronidase nanoparticles to degrade the ECM to enhance tumor penetration [37,38]. Recently, Hong et al. reported an exosome nanoparticle with active PH20 hyaluronidase (Exo-PH20), which can transport cargos into the tumor through hyaluronan degradation. Exosomes are relatively advantageous because of their endogenous origin and their potential functions for intercellular communication, which enhance tumor therapy and drug delivery. Hence, the exosome-mediated delivery of doxorubicin efficiently enhances drug distribution in the deep tumor and inhibits tumor growth efficiently [39].

Similar concepts were also reported with losartan to decrease collagen I barriers in the dense tumor ECM and enhance tumor penetration [40,41]. Specifically, Parodi et al. used an enzymatic complex, bromelain, to integrate cysteine and sulfhydryl proteases (the papain family). Bromelain is able to cleave the peptide sequence Bz–Arg–Arg–*p*-nitroanilide and is applied to tenderize meat by proficiently digesting the ECM. Endowed with this property, bromelain-conjugated mesoporous silica nanoparticles were shown to increase the digestion and diffusion into the tumor and achieve tumor penetration by ECM disruption [42].

In addition to degradation, damage to the matrix by hyperthermia and external stimuli is another common method of eliminating the dense tumor ECM. He et al. developed a deep tumor-penetrating photothermal nanohybrid loaded with a near-infrared (NIR) agent to suppress tumor and cancer metastasis. The lipophilic NIR agent, 1,1-dioctadecyl-3,3,3,3-tetramethylindotricarbocyanine iodide (DiR), which possesses excellent light-absorbing ability, was loaded into polymeric micelles composed of poly(ethylene glycol)-block-poly(2-diisopropylmethacrylate). The DiR-labeled polymeric particles exhibited excellent photothermal conversion efficiency and a narrow size distribution with a mean diameter of approximately 24.5 nm. When irradiated by the 808-nm laser, the particles generated stronger heat energy than free DiR. Furthermore, both the cell proliferation and migration of metastatic cancer cells were significantly reduced by the particles with NIR irradiation. Moreover, the particles achieved strong tumor penetration, which was actuated by single NIR irradiation [43].

Our group also indicated that mesoporous iron-oxide carriers can deliver therapeutic cargos by penetrating deep into the tumor via applying an external high-frequency magnetic field (HFMF) at the targeted site. A gasified molecule, perfluorohexane (PFH), and a hydrophobic anticancer drug, paclitaxel, were encapsulated into the carriers, and the local bursting gas generation and drug release were actuated through the HFMF treatment, where the PFH exhibited a phase-transition temperature at 56 °C. After a short-duration HFMF treatment, the gasification of PFH substantially ruptured the three-dimensional tumor spheroids in vitro and improved tumor penetration. Our work combines HFMF-induced PFH gasification and hyperthermia to promote the deep penetration for tumor damage and inhibition [44].

In addition to NIR and HFMF, pulsed focused ultrasound was used by Lee et al., who employed a modified ECM approach, to improve the tumor penetration of therapeutic agents. They demonstrated that the tumor penetration of intravenously injected nanoparticles in mice was greatly inhibited by collagen and hyaluronan components surrounding the tumor. To overcome the obstacle of the tumor barrier, the ECM of the tumor cells was treated by focused ultrasound with a relatively low power density (20 W/cm^2^). After the noninvasive ultrasound treatment, the ECM of the tumor tissue remodeled and degraded, increasing blood flow and therapeutic agents in the deep tumor. Furthermore, the increase in therapeutic agents at the tumor was 2.5-fold that of untreated tumors [45]. In brief, by combining outer stimuli such as NIR, HFMF, or ultrasound with certain nanomaterials or by degrading specific proteins or compounds, the dense tumor extracellular matrix can be destroyed, which increases the penetration of the tumor and the delivery of the functional nanoparticles.

### 4.2. Vascular Disruption

In addition to the dense extracellular matrix of the tumor, the blood vessels in the tumor can also block nanoparticle penetration. Even though the EPR effect results from leaky blood vessels, if the size of the nanoparticles is too large to cross the blood vessels, there is another obstacle for tumor penetration. To overcome the barriers, Liu et al. used combretastatin A4 (CA4), a vascular disrupting agent, to damage and disrupt the vessels. They developed a poly(l-glutamic acid)-CA4 conjugate (PLG-CA4) nanohybrid to improve the drug/particle accumulation and retention at the targeted tumor site, leading to a homogeneous distribution and gradual release. In the tumor microenvironments, the hybrids caused the vascular disruption effectively and enhanced the therapeutic efficiency. Furthermore, with only one treatment of the hybrids, persistent vascular disruption of the tumor was observed after 72 h in a tumor-bearing mouse model. The tumor suppression also showed that PLG-CA4 has a tumor suppression rate of 74%, which is a clear improvement when compared to that of commercial vascular agents that exhibit a tumor suppression rate of approximately 24%. This study provided a new approach to penetrate the tumor through disrupting blood vessels [46].

On the other hand, Ho et al. developed a nanodroplet with encapsulated doxorubicin for penetrative chemotherapy. By applying a physical acoustic droplet vaporization method, intravital and ultrasonic imaging was observed in real time in vivo. Furthermore, the anticancer drug, doxorubicin, was perfused into the tumor after the treatment. The histological analysis suggested clear morphological changes in the tumor, which also indicated dimensional permeation and homogeneous drug distribution. This approach combined both physical anti-vascular treatments and chemotherapy to enhance drug penetration and possibly elicit immune responses at the tumor. The excellent intratumoral distribution of the drug improved the therapeutic efficacy by both resisting the tumor growth and increasing the animal survival rate [47]. Another strategy of vascular disruption is inducing by micro- or nanoscale droplets (MD or ND) actuated by ultrasound sonication to improve drug penetration at the tumor. Upon the application of an acousto-optical treatment comprising a 2-MHz focused ultrasound transducer in a window-chamber mouse model, liposomes exhibited tissue penetration. Compared to the MD and ND treatments, the doubling of tumor accumulation was observed through the focused ultrasound enhancement. The main mechanism of the accumulation enhancement was induction by vascular disruption caused by acoustic droplet vaporization, leading to strong tumor penetration. In addition, a longer lifetime was detected in the ND group than in the MD group, suggesting the potential vaporization and conversion of bubble cavitation by the ND. These behaviors all contributed to the disruption of tumor vessels to improve particle penetration and transport [48].

Another group used ultrasound to enhance tumor penetration via disrupting the tumor vasculature. Park et al. developed dynamic contrast-enhanced magnetic resonance imaging by applying focused ultrasound (FUS)-induced permeability, drug delivery, and drug retention in a brain tumor. They investigated the deformation of particles and the concentration variation of an anticancer drug (doxorubicin). After implantation of the glioma, they used 690-kHz ultrasound and microbubbles to induce the FUS to disrupt the BBTB in the tumors. Afterward, the dose of doxorubicin was shown to be 5.67 mg/kg. The levels of FUS-induced BBTB disruption were clearly higher than that of the control group after sonicating the tumors. This demonstrates that FUS- and microbubble-enhanced doxorubicin transport through the BBB and BBTB was successful. Such improved retention enhances the potency of the anticancer drug. Furthermore, with FUS irradiation, drug penetration and retention may both be improved [49].

Unfortunately, in recent years, chemical vascular disrupting agents used clinically exhibited potential toxicity concerns. For example, combrestatin, as mentioned above, showed dose-limiting side effects of pulmonary embolism and coronary vasospasm when used in human trials. Thus, in addition to using ultrasound-induced vascular disruption, Kunjachan et al. revealed that the tumor neovasculature is also a critical target for radiation therapy, which can be used for vascular disruption. They demonstrated that functional gold nanoparticles both decrease off-target effects and enhance local accumulation at the targeted tumor site. Furthermore, the functional gold particles specifically damage the tumor vessel when subjected to radiation without the use of any toxic vascular disrupting agents. This two-fold targeting strategy of vascular disruption and tumor penetration minimizes the normal tissue toxicity and significantly boosts therapeutic efficacy [50].

## 5. Tumor Penetration by Combinational Strategies

With the combination of different strategies, tumor penetration can be achieved more comprehensively and more easily. As functional nanoparticles arrive at the tumor site by the EPR effect due to tumor heterogeneity, the functional nanoparticles not only increase the drug distribution, but also specifically and homogeneously treat the tumor. Therefore, multiple functionalizations can be used to conquer the various obstructions in the tumor, which would enhance tumor penetration and drug delivery.

### 5.1. Size and Ligand Functionalization

Combining size changeability with ligand functionalization, Kim et al. developed pH-sensitive nanohybrid particles composed of gold nanoparticles and a packed DNA assembly. They modified the surface with B-cell lymphoma 2 (Bcl-2) antisense and i-motif binding sequences. Each DNA sequence was hybridized via the Bcl-2 antisense and i-motif as the functionalization ligands, and DNA clustering of the gold nanoparticles provided tumor targeting for A549 cells and drug-loading capabilities. For size changeability, the anticancer agent was released by deforming the structures while changing the pH in the tumor microenvironment and in the late endosome. The antiapoptotic Bcl-2 protein was also affected by the antisense-conjugated gold particles, which resulted in drug-mediated apoptosis. The function of this tumor-homing DNA nanocluster as a penetrating drug delivery and controlled drug release system was evaluated [51].

Ruan et al. also reported a novel nanohybrid with a targeting ligand (RGD) and size transition via MMP-2 degradation to inhibit tumor growth. Their nanohybrid was fabricated by mixing gold nanoparticles with matrix metalloproteinase-2 (MMP-2)-degradable gelatin nanoparticles. The anticancer drug doxorubicin was conjugated to the particle through a pH-sensitive hydrazone bond. In addition, RGD, which specifically targets α_v_β_3_ receptors, was used to enhance tumor targeting. For tumor penetration, the size transition of the particles was actuated from 185.9 to 71.2 nm after incubating with MMP-2, and doxorubicin was released in a pH-dependent manner. The in vivo study indicated that the particle actively targeted the 4T1 tumor by ligand functionalization and penetrated through the dense ECM by size changeability and collagen diffusion, achieving enhanced accumulation and tumor penetration into the deep tumor region [52].

**Figure 5:**
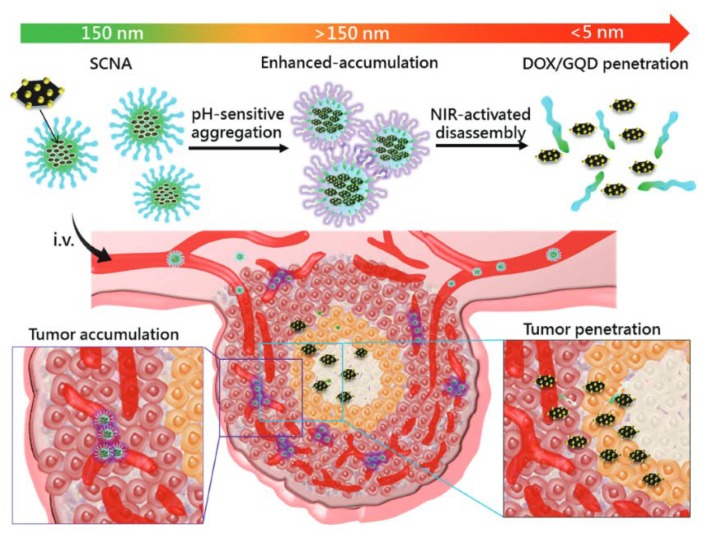
Combinational strategies for tumor penetration. Our group developed tumor penetration with two steps. First, the SCNAs transported drug-loaded GQD to the tumor, and the size of the SCNAs increased in the weakly acidic tumor environment to enhance local accumulation. Second, light-actuated delivery of GQDs and drug-enhanced tumor penetration. Reprinted from Reference [53] with permission from John Wiley and Sons, 2017.

Our group also generated a size-changeable graphene quantum dot (GQD) nanoaircraft (SCNA) that possesses hierarchical tumor-targeting efficacy and transports a high dose of therapeutic agents into deep tumors. The particle was fabricated by ultrasmall GQDs and a pH-responsive polymer that aggregates in the weakly acidic microenvironment of the tumor and is able to stabilize at neutral pH and function as a ligand. The size transition in the tumor microenvironment is triggered by an external near-infrared irradiation with a wavelength of 808 nm, which releases GQDs loaded with anticancer drugs to the deep tumor and facilitates drug transportation in the tumor via the ultrasmall materials. Furthermore, the GQD and drugs can affect the neighboring cancer cells for wide-range cancer cell killing. The hierarchically targeted particle can be achieved by pH-sensitive and light-triggered penetrated delivery of drugs by size changeability into the deep tumors [53] (Figure 5).

**Figure 6:**
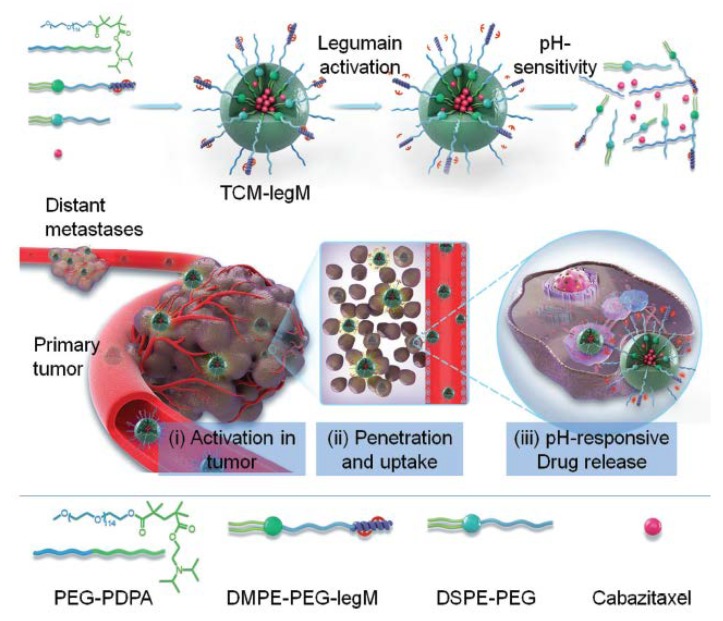
Combinational strategies for tumor penetration. He et al. demonstrated their TCM-legM as functional nanoparticles: (**i**) specific activation targeting by the highly expressed legumain protease to the tumor; (**ii**) improving tumor penetration and cell internalization; (**iii**) actuating the weak acid-responsive drug release to achieve antimetastatic therapy. Reprinted from Reference [54] with permission from John Wiley and Sons, 2018.

Recently, He et al. developed tumor-microenvironment-activated micelles loaded with cabazitaxel, an anticancer drug, that were modified by legumain-specific melittin for pH-triggered drug release in weakly acidic microenvironments; these micelles were named TCM-legM nanoparticles for treating breast cancer. The TCM-legM nanohybrid is stable in blood circulation and can be actuated by the highly expressed legumain in the tumor to enhance the targeting effect and tumor penetration. Thereafter, the TCM-legM internalized by the cancer cells rapidly releases the encapsulated drug via conformational changes of the micelles in the weakly acidic environment for anticancer metastasis therapy. TCM-legM significantly inhibits the proliferation and migration of metastatic breast cancer cells. In addition, TCM-legM transports the anticancer drug to deep regions of the primary and metastatic tumors through the mechanism of effective cell internalization, leading to 93% tumor suppression in lung tumor [54] (Figure 6).

### 5.2. Ligand Functionalization and Modulation

For the association of ligand functionalization and the modulation of the tumor microenvironment, our group reported a sponge-like graphene nanosheet (termed graphene nanosponge) coated with lipid bilayers as a photothermal particle for tumor damage. Upon NIR irradiation, the particles release the anticancer drug, docetaxel (DTX), and generate gas by gasifying perfluorohexane (PFH) locally. Conjugated with lactoferrin (Lf), the particles exhibit good tumor-targeting effects and transcytosis in the tumor, leading to increased tumor accumulation and penetration. In addition, the integration of two therapeutic chemicals (DTX and PFH) in one transport particle is able to generate heat and gas and destroy the dense ECM of the tumor to suppress the tumor within a few minutes of the NIR treatment. Furthermore, no tumor recurrence is detected after 60 days. The graphene nanosponge is an outstanding drug and energy transportation platform for tumor penetration and combination therapy of tumor [55].

On the other hand, using a well-known ligand RGD, Wang et al. used iRGD-conjugated particles for dual transportation of a photosensitizer, indocyanine green (ICG), and a hypoxia-activated prodrug, tirapazamine (TPZ), to the tumor. The iRGD-conjugated particles exhibited enhanced penetration in an ex vivo tumor model and in in vivo breast tumors via the mechanism of RGD ligand transcytosis. NIR irradiation induced the photodynamic therapy of ICG and triggered the tumor suppression of the codelivered TPZ for a synergistically therapeutic effect. Both primary and metastatic tumors were significantly reduced by the iRGD-conjugated particles with low side effects. The combination of transporting ICG and TPZ in a compact particle showed better therapeutic effects when compared to particles mixed with individual drugs. The study exhibits the strong therapeutic effect of iRGD in tumor penetration for combination therapy and hypoxia-modulated drug therapy [56].

Recently, Zhao et al. demonstrated that photoresponsive particles can increase the effect of photodynamic therapy (PDT) through generating oxygen locally for normalized ROS environments in the tumor. After the poly(ethylene glycol)-poly(ε-caprolactone) copolymers were emulsified, double-emulsion particles (W/O/W) were formed. The particles encapsulated both a photosensitizer (IR780) and an oxygen depot (perfluorooctyl bromide (PFOB)) in the oil and water phases in the particle. Furthermore, the peptide c (CRGDK) was conjugated onto the particles to provide tumor-penetrating ability and enhance the accumulation of the particles in both blood vessels and hypoxic regions of the tumor. Once PFOB was released in the hypoxic region, hypoxia was alleviated, and the effect of PDT was modulated. By ligand functionalization and modulation of the tumor microenvironment, the tumor distribution of IR780 was enhanced, and the provided oxygen amplified the therapeutic effect of the cancer cells on PDT efficiently [57].

### 5.3. Size and Modulation

For size switchability that is associated with that modulation of that tumor microenvironment, Ju et al. reported a nanogel, which is able to change its swelling property in intra- and intercellular conditions to achieve tumor penetration and treatment. The gel consists of three parts: *N*-lysinal-*N*′-succinyl chitosan, poly(*N*-isopropylacrylamide) (PNIPAM), and bovine serum albumin (BSA). When the pH is approximately 6.0, the optimized synthesized *N*-lysinal-*N*′-succinyl chitosan exhibits a charge-switching property. The core of the nanogel is constructed by the polymerization of NIPAM in neutral conditions. Furthermore, BSA is coated on the surface of the nanogel to maintain stability in the blood and exhibits pH-sensitive swelling properties. The nanogel possesses a negative charge at pH 7.4 and is able to enhance the EPR effect at the tumor site after intravenous administration; afterward, it is internalized into endosomes and lysosomes by the cancer cells in the peripheral tumor. At the low pH value of the endosome (pH 5.0–6.0) or lysosome, the amino groups of the nanogel are protonated rapidly to actuate the electrostatic repulsion of the nanogel, as well as to induce the swelling of the core to release the encapsulated anticancer drugs. By modulating the tumor cells, the swelling and charge-switching of the nanogel causes the endo-lysosomal burst release, facilitating the repeated transportation of the gel into the cytosol. The nanogel shrinks and is liberated from the dead cells, thereby infecting the neighboring cancer cells close to the tumor center. The sequentially repeated intercellular delivery system combined with the size effect and modulation of the tumor microenvironment provides a potential platform for deep tumor penetration of nanoparticles [58].

In addition to therapy and imaging applications, Zhou et al. reported a magnetic droplet vaporization technique for tumor theranostics that can be controlled by an external magnetic field. The gasifiable perfluorohexane (PFH) is loaded in the iron-oxide particles. The magnetic particles can be triggered by an external alternating current (AC) magnetic field to rapidly generate the intense heat and produce hyperthermia around the tumor tissue. The embedded PFH with a boiling point of 56 °C can be gasified to improve the function of ultrasound imaging by changing the size of the small microbubbles. The magneto-thermal conversion further ablated the tumors by hyperthermia, which increased tumor penetration and tumor imaging [59].

On the other hand, Chen et al. also synthesized multifunctional pH-/H_2_O_2_-responsive human serum albumin (HSA)–MnO_2_ nanoparticles using the albumin-based mineralization approach. Upon systemic injection into the tumor by the EPR effect, the HSA–MnO_2_ nanoparticles reacted with endogenous H_2_O_2_ inside the tumor to generate oxygen and regulate the hypoxic region of tumors, overcoming hypoxia-mediated photodynamic resistance for tumor modulation. For size switchability, the reaction of MnO_2_ with H^+^ and H_2_O_2_ in the tumor microenvironment resulted in the gradual disassembly of HSA–MnO_2_ into albumin complexes with sub-10-nm sizes, achieving notably enhanced intratumoral penetration. The size of the smart particles responded to the tumor microenvironment; intratumoral penetration was increased and the tumor microenvironment was simultaneously modulated [60].

Recently, Hu et al. also presented a well-developed nanohybrid able to respond to the tumor microenvironment, penetrate into the tumor, and achieve combination therapy. The smart hybrid is composed of hyaluronic-acid shells, a light-sensitive nitric-oxide donor, and an anticancer drug, and indocyanine green (ICG) serves as the photothermal agent. The particle demonstrates excellent tumor penetration through the enzymatically degradable shells and laser-improved photopenetration effects upon NIR irradiation. The in vivo therapeutic effects of the particles exhibit anticancer efficiency under the NIR irradiation. Hence, the validity of their study also provides a novel tactic for functional hybrids to regulate the tumor microenvironment for drug delivery and to decrease the heterogeneity of anticancer drugs in the tumor [61].

### 5.4. RNA Nanotechnology

RNA agents such as small interfering RNA (siRNA) and messenger RNA (mRNA) exhibit potential in displaying the functionality of specific gene alterations and enabling therapies for cancer treatment. Despite the advancements of RNA, the safe and effective delivery of RNA to targeted site remains a challenge in clinical applications. To overcome the obstacles of in vivo siRNA delivery, various theranostic nanoparticles have been designed and prepared. Xu and co-workers reported that oligoarginine-functionalized polymers with sharp pH-response can encapsulate siRNA and perform the feature of long blood circulation and pH-responsive endosomal membrane penetration [62]. Conjugating to a targeting ligand, this platform efficiently binds prostate-specific membrane antigen-expressing cells and silences target gene expression. Furthermore, Lee and his colleagues developed a polymeric siRNA nanoflower by rolling circle transcription method to amplify antisense strands of siRNA and to anneal its chimeric RNA-DNA sense strands, revealing superior suppression in tumor growth [63]. Moreover, self-assembled intertwining DNA-RNA nanocapsules transported DNA CpG and short hairpin RNA (shRNA) adjuvants synergistically, exhibiting significant inhibition in the progression of neoantigen-specific colorectal tumors [64].

## 6. Conclusions

In summary, much effort and many excellent results were revealed for promoting the penetration of functional nanoparticles into deep tumors and enhancing the therapeutic efficacy for cancer treatments (Table 1); there will be great challenges in translating these from the bench to the bedside. Therefore, these novel nanoparticles should be optimized comprehensively. In addition, the heterogeneity of solid tumors is complicated and needs to be further elucidated for the concept of precision medicine. For example, the tumor microenvironment and physiological characteristics of each patient are highly variable, which results in inconsistent therapeutic efficacy. Although several nanomedicines were approved by the FDA for treating cancer patients, the impact of hindering nanohybrid permeability may be a forward-looking process for clinical trials and marketing. Therefore, the basic research of tumor biology related to nanomedicine should be intensely studied, which can provide guidance on the design and fabrication of functional nanoparticles for clinical trials.

On the other hand, the safety of nanomedicine should be thoughtfully investigated. As multiple components are constructed in the nanoparticles, the bio-related factors such as biostability, biocompatibility, and biodegradability of these therapeutic nanomedicines should be carefully studied. Thus, the design of nanoparticles should be fabricated to be as simple as possible in accordance with the clinical applications. With the strategies of size changeability, ligand targeting, and modulation of the tumor microenvironment, nanohybrid penetration for improving the tumor treatment was explored more completely. We look forward to the development of new drug-delivery systems and the translation to clinical trials, as more effective nanomedicine will be on the market for cancer patients in the future.

## Figures and Tables

**Table 1:** Integrations of functional nanoparticles with size changeability, ligand functionalization, modulation of tumor microenvironment, and combinational strategies for penetrated delivery for tumor treatments. MMP: matrix metalloproteinase; RGD: arginine–glycine–aspartic acid; ECM: extracellular matrix; CRGDK: Cys–Arg–Gly–Asp–Lys; PFOB: perfluorooctyl bromide.

Strategy	Nanoparticles	Penetrative Tactics/Targets	Therapeutic Remarks	Ref
Size Changeability	Spiropyran nanoparticle	UV light	Docetaxel delivery for HT-1080 tumors	[12,13]
	Micelle-loaded nanocapsule	Near-infrared	Docetaxel delivery for RG2 tumors	[14]
	CO_2_ generating nanoparticle	Ultrasound	Docetaxel delivery for SCC7 tumors	[15]
	NO generating liposome	Ultrasound	Doxorubicin delivery for MCF-7/MIAPaCa-2	[16]
	Magnetic nanocapsule	Magnetic field	Camptothecin delivery for MT2 tumors	[17,18]
	Gelatin nanoparticle	MMP-2, MMP-9	Doxorubicin delivery for 4T1 and B16F10	[19,20]
	MMP-cleavable nanoparticle	MMP	Paclitaxel and siRNA for 4T1 tumors	[21]
	DNase-degradable nanoclew	pH value	Doxorubicin delivery for MCF-7 cells	[22]
	Dendrimeric nanobomb	pH value	Cisplatin delivery for BxPC-3 tumors	[23,25]
	Hypoxia-responsive lipid	oxygen concentration	Gemcitabine delivery for BxPC-3 tumors	[26]
	Redox-sensitive lanthanide	Reductive condition	Peptides delivery for HCT116 tumors	[27]
Ligand Functionalization	Albumin nanoparticle	Albumin-binding protein	Paclitaxel delivery for U87 tumors	[28]
	IF7 nanoparticle	Specific biomarker Anxa1	Paclitaxel delivery for MCF-7/ADR tumors	[6,29]
			SN38 delivery for HCT116 tumors	
	RGD polymer/ interleukin-13	αvβ3/αvβ5 integrin	Oxaliplatin delivery for U87 tumos	[31,32]
	iRGD lipoprotein nanoparticle	αv integrin & neuropilin-1	Paclitaxel/Indocyanine green delivery for A549	[33]
	Tf nanoparticle	Tf receptor	Paclitaxel delivery for C6 tumors	[34]
	Lf/tLyP-1 nanoparticle	Lf receptor & neuropilin-1	Paclitaxel delivery for C6 tumors	[35]
	Monocyte nanoparticle	Chemokines	Doxorubicin delivery for Tramp-C1 tumors	[36]
Modulation of tumor	HAase nanoparticls	Hyaluronan in ECM	Chlorine e6 delivery for 4T1 tumors	[37,38]
			Doxorubicin delivery for 4T1 tumors	
	PH20-modified exosomes	Hyaluronan in ECM	Doxorubicin delivery for PC-3 tumors	[39]
	Losartan nanoparticle	Collagen in ECM	Paclitaxel delivery for 4T1 tumors	[40,41]
	Bromelain nanoparticle	Collagen in ECM	Silica delivery for MDA-MB-231 tumors	[42]
	Photothermal nanoparticle	Damaged ECM	Photothermal DiR for 4T1 tumors	[43]
	Magne-thermal nanoparticle	Damaged ECM	Perfluorohexane/Paclitaxel delivery for RG2	[44]
	Pulse-HIFU nanoparticle	Loosened ECM	Chitosan delivery for SCC7 tumors	[45]
	Combretastatin-A4 NP	Vascular disruption agent	Combretastatin-A4 for C26 tumors	[46]
	Droplet vaporization NP	Damaged vascular	Doxorubicin delivery for Tramp-C1 tumors	[47,48]
	Focused ultrasound Gd-DTPA	Loosened vascular	Doxorubicin delivery for blood brain barrier	[49]
	Radiation responsive NP	Damaged vascular	AF647 delivery for Panc-1 tumors	[50]
Size + Ligand Functionalization	ODNs nanoparticle	pH value + Tumor-homing	Doxorubicin delivery for A549 tumors	[51]
	Gelatin + RGD nanoparticle	MMP-2 + αvβ3 integrin	Doxorubicin delivery for 4T1 tumors	[52]
	GQD-loaded nanoparticle	Near-infrared + pH value	Doxorubicin delivery for RG2 tumors	[53]
	TCM-LegM nanoparticle	pH value + Legumain	Cabazitaxel delivery for 4T1 tumors	[54]
Ligand Functiona-lization + Modula-tion of tumor	Lf + Photothermal NP	LfR + Damaged ECM	Perfluorohexane/Docetaxel delivery for RG2	[55]
	iRGD + Tirapazamine	αv integrin + Hypoxia	Indocyanine green/Tirapazamine for 4T1 tumors	[56]
	CRGDK + PFOB	neuropilin-1 + Hypoxia	PFOB delivery for MDA-MB-231 tumors	[57]
Size + Modulation of tumor	Swelling–shrinking NP	pH value + Lyso bursting	Doxorubicin delivery for HepG2 tumors	[58]
	Magnetic + Droplet vapor	Magnetic field + Vascular	Perfluorohexane delivery for MDA-MB-231	[59]
	HSA-MnO_2_ nanoparticles	pH value + Hypoxia	Doxorubicin/Indocyanine green for 4T1 tumors	[60]
	Hyaluronan/NO/ICG NP	HAase + Near-infrared	Chlorine e6 delivery for 4T1 tumors	[61]
